# Individualized breastfeeding support for acutely ill, malnourished infants under 6 months old

**DOI:** 10.1111/mcn.12868

**Published:** 2019-08-02

**Authors:** Martha Mwangome, Sheila Murunga, Jane Kahindi, Prinilla Gwiyo, Grace Mwasho, Alison Talbert, Laura Kiige, Betty Samburu, Neema Mturi, Amina Abubakar, Caroline Jones, James A. Berkley

**Affiliations:** ^1^ Kenya Medical Research Institute (KEMRI)/Wellcome Trust Research Programme Kilifi Kenya; ^2^ Childhood Acute Illness and Nutrition (CHAIN) Network Nairobi Kenya; ^3^ Department of Nutrition Kilifi County Hospital Kilifi Kenya; ^4^ United Nations Children's Fund (UNICEF) Kenya County Office Nairobi Kenya; ^5^ Nutrition and Dietetics Unit, Family Health Division Ministry of Health Nairobi Kenya; ^6^ Institute of Human Development Aga Khan University Nairobi Kenya; ^7^ Centre for Tropical Medicine and Global Health, Nuffield Department of Clinical Medicine University of Oxford Oxford UK

**Keywords:** acute malnutrition, exclusive breastfeeding, infants under 6 months, peer supporters

## Abstract

Reestablishing exclusive breastfeeding is the cornerstone of the 2013 World Health Organization (WHO) treatment guidelines for acute malnutrition in infants less than 6 months. However, no studies have investigated guideline implementation and subsequent outcomes in a public hospital setting in Africa. To facilitate implementation of the WHO 2013 guidelines in Kilifi County Hospital, Kenya, we developed standard operating procedure, recruited, and trained three breastfeeding peer supporters (BFPS). Between September 2016 and January 2018, the BFPS provided individual breastfeeding support to mothers of infants aged 4 weeks to 4 months admitted to Kilifi County Hospital with an illness and acute malnutrition (mid‐upper‐arm circumference < 11.0 cm OR weight‐for‐age *z* score < −2 OR weight‐for‐length z score < ‐2). Infants were followed daily while in hospital then every 2 weeks for 6 weeks after discharge with data collected on breastfeeding, infant growth, morbidity, and mortality. Of 106 infants with acute malnutrition at admission, 51 met the inclusion criteria for the study. Most enrolled mothers had multiple breastfeeding challenges, which were predominantly technique based. Exclusive breastfeeding was 55% at admission and 81% at discharge; at discharge 67% of infants had attained a weight velocity of >5 g/kg/day for three consecutive days on breastmilk alone. Gains in weight‐for‐length z score and weight‐for‐age z score were generally not sustained beyond 2 weeks after discharge. BFPS operated effectively in an inpatient setting, applying the 2013 updated WHO guidelines and increasing rates of exclusive breastfeeding at discharge. However, lack of continued increase in anthropometric *Z* scores after discharge suggests the need for more sustained interventions.

**Key messages**

- Lack of evidence on the feasibility and outcome of implementing the WHO (2013) nutritional rehabilitation guidelines to treat ill, malnourished infants u6m has slowed their application.
- Well‐trained and supervised Breastfeeding Peer Supporters (BFPS) operating in an inpatient setting can implement the WHO (2013) guidelines for nutritional rehabilitation of ill, malnourished infants u6m effectively, enhancing EBF from 55 to 81% with 67% of all infants reaching the recommended weight velocity on breastmilk alone by discharge.
- Lack of continued increase in anthropometric *Z* scores after discharge suggests the need for more sustained approaches to improve growth and survival after discharge.

## 1 INTRODUCTION

Acute malnutrition among infants aged below 6 months (u6m) is increasingly recognized as an important public health problem. Globally, it is estimated that 4.7 million infants u6m are severely wasted (weight‐for‐length z score [WLZ] < ‐2) and an additional 3.8 million are moderately wasted (Kerac et al., 2011). The risk of mortality associated with acute malnutrition is higher in infants aged u6m than among children aged above 6 months (Grijalva‐Eternod et al., 2017). Infants are likely to become malnourished if they are suboptimally breastfeeding, have an acute infection or congenital abnormality disrupting their appetite, and ability to breastfeed or are either born prematurely, small for gestational age or as a twin (Bhutta et al., 2013; Thurstans, 2015).

In 2013, the World Health Organization (WHO) revised the guidelines for identifying and treating acute malnutrition in children, including for the first time recommendations on how to identify and manage acute malnutrition in infants u6m (World Health Organization [WHO]., 2013). The updated guidelines focus on inpatient reestablishment of exclusive breastfeeding (EBF; WHO., 2013). This approach differs from the nutrition rehabilitation guidelines for older children for whom the use of Therapeutic formulae (F75 and 100) or ready to use therapeutic food is recommended.

Currently in most low‐ and middle‐income settings, the recommendations to reestablish EBF are inconsistently applied (Vygen, Roberfroid, Captier, & Kolsteren, 2013) and there is limited evidence on the outcomes of guideline implementation. Programmatic reports (Corbett, 2000; Oberlin & Wilkinson, 2008) and two published studies (Seema Patwari & Satyanarayana, 1997; Singh, Rai, Mishra, Maurya, & Srivastava, 2013) suggest that reestablishing EBF among inpatient acutely malnourished infants u6m has variable success (29 to 92%). Breastfeeding peer supporters (BFPS) have been shown to increase the prevalence of EBF among mothers in the community in low resource settings (Ara et al., 2018; Chola et al., 2015; Kaunonen, Hannula, & Tarkka, 2012; Ochola, Labadarios, & Nduati, 2013; Tylleskar et al., 2011), but little is known about the acceptability or effectiveness of this approach for enhancing EBF for hospitalized malnourished infants. Additionally, other than Oberlin and Wilkinson, 2008, no studies have followed‐up infants u6m after discharge. Consequently, there is limited evidence on the effectiveness of BFPS in a hospital setting, or the maintenance of EBF postdischarge, or whether EBF is sufficient to support adequate growth and sustain catch‐up growth in malnourished infants u6m.

We set out to use BFPS to facilitate the implementation of the 2013 WHO guidelines among hospitalized malnourished infants u6m in rural Kenya, assessing the feasibility of using this approach and evaluating outcomes of effective guideline implementation. This paper describes the process of guideline implementation and reports on the outcome: EBF, infant growth, morbidity, and mortality up to 6 weeks' postdischarge.

## 2 METHODS

### 2.1 BFPS selection and training

Recruitment of three BFPS was initiated through a job advertisement placed on the Kilifi county hospital's notice boards. Selection criteria included being mothers with breastfeeding experience who were members of the hospital catchment community and of similar age, culture, and social status to the mothers whose infants u6m were admitted to Kilifi County Hospital (KCH) with malnutrition. The BFPS were all literate but had no college training or formal health work experience. Alongside other paediatric ward‐based health workers (nurses, clinical officers, and paediatricians), the selected BFPS underwent a 5‐day introduction to lactation management training, which was designed and delivered by the maternal, infant, and young child nutrition experts from United Nations Children's Fund (UNICEF) and Ministry of Health Kenya. The training drew on existing WHO, UNICEF, and Ministry of Health breastfeeding materials (United Nations Children's Fund [UNICEF], 2013; United Nations, 1986; WHO, 1995, 1999, 2005, 2010a, 2010b; WHO & UNICEF, 2006, 2016; WHO & UNICEF., 2009; WHO., 2013) adapted to facilitate the implementation of the 2013 WHO guidelines. The training programme including content and sources of the training material is shared in Table S1 in the Supporting Information.

### 2.2 Guideline implementation

A standard operating procedure (SOP) for WHO guideline implementation was developed by the team of investigators (Figure S1 in the Supporting Information). The purpose of the SOP was to ensure consistent guideline implementation, and it was designed to address three questions: (a) What activities are needed to meet the recommendations? (ii) Who should be responsible for implementing these activities? (iii) How will outcomes be evaluated and documented? Key breastfeeding support activities and procedures were identified, and tools for implementing and evaluating breastfeeding support were drawn from existing WHO and UNICEF breastfeeding support manuals (Ministry of Health, 2007, 2016; WHO, 1989, 1995, 2010b; WHO et al., 2001; WHO & UNICEF, 2016). The final SOP included three main steps.

Step 1: Detailed breastfeeding assessment with identification of breastfeeding challenges to be carried out by the paediatric nutritionist (PN) accompanied by the BFPS within 48 hr of admission. Based on the assessment, a lactation plan was drawn‐up, prioritizing support activities to increase breastmilk quantity and improving breastfeeding technique, with individualized plans for each mother. These individual plans were subsequently systematically implemented by the BFPS.
Step 2: Review of the lactation plan and its effect on breastmilk quantity and infant weight gain. The review was led by the PN and was undertaken on the third or fourth days after admission (and each subsequent third or fourth day during admission until step 3). The review involved (a) checking if the lactation plan had been fully implemented, (b) assessing whether the mother was producing more or any breastmilk, (c) gauging whether the infant was consistently gaining weight, and (d) gaining insights from the BFPS on breastfeeding challenges experienced by the mother. Information collected during the review was used to assess if revisions to the lactation plan were required. If the review found that the mother was successfully exclusively breastfeeding, then monitoring of the velocity of weight gain over a 3‐day period was incorporated into the plan and the infant was ready to progress to step 3. The recommendations from the review were incorporated into a revised plan (where required) that was subsequently implemented by the BFPS.
Step 3: Review of progress for discharge. This third step was led by the PN usually a week or so after admission and involved the infant being moved onto growth velocity monitoring for at least 3 days. Infants were weighed every morning, so it was possible to calculate their daily growth velocity. Infants observed to have a weight velocity of >5 g/kg/day for three consecutive days when fed on breastmilk alone, were considered nutritionally rehabilitated and fit for discharge as per the WHO discharge criteria. Participants were considered to be in the lactation failure category if they had received breastfeeding support for 14 days without meeting the discharge criteria (Figure S1 **)**.

### 2.3 Study site

The IBAMI study was implemented in the paediatric ward of the KCH, a level 4 hospital providing primary and inpatient care and located in coastal rural Kenya. Kilifi is one of the poorest counties in Kenya with >40% of the population living below the poverty line (Kenya National Bureau of statistics, 2015). Each year, approximately 150 infants aged below 6 months with acute malnutrition (WLZ < ‐2) are admitted to KCH paediatric ward.

### 2.4 Study design

This was a prospective cohort pilot study enrolling malnourished infants aged between 1 and 4 months admitted to KCH hospital with an illness. The primary outcome was the successful implementation of the 2013 WHO updated guidelines for inpatient treatment of malnourished infants. Secondary outcome included providing a description of the cohort's mortality and morbidity as well as growth during admission and after discharge.

### 2.5 Study population and procedures

Only infants admitted to KCH with a low anthropometry (mid‐upper‐arm circumference [MUAC] < 11.0 cm OR weight‐for‐age *z* score [WAZ] < ‐2 OR WLZ < ‐2) and an illness were considered eligible for enrolment. Infants without a possibility to breastfeed and those diagnosed with a congenital malformation were excluded from the study.

#### 2.5.1 Identification

We developed a three stage screening procedure. At KCH anthropometry and age is routinely collected at admission and absolute measures of weight, MUAC, length, and head circumference are immediately entered into a computerized platform known as the Kilifi integrated data management system (KIDMS). Within the KIDMS, *Z* scores are automatically generated using the WHO 2006 growth references categories (WHO, 2006). For this study, the KIDMS was set up to flag infants presenting within the required age and nutritional status as the first screening stage. Data on congenital malformation are not routinely collected in the KIDMS so as a second screening stage, criteria were developed by the study for the admitting clinicians to indicate, in the KIDMS, infants with congenital malformations including cleft lip and palate, congenital heart disease, and dysmorphic features (exclusion criteria). The third stage involved assessing caregiver's possibility and willingness to breastfeed. A three‐question criteria specifically developed for this study was applied.

#### 2.5.2 Recruitment

Following the screening process, the mothers of potentially eligible infants were informed about the study and asked whether or not they would be willing to participate. Written consent was obtained from those who agreed to participate and their infants were given a study number and a study file was opened for them. On recruitment, information about the infant's current breastfeeding status, place of birth, birth weight, gestational age at birth, birth complications, infection history, immunization status, and sibling order were collected. In addition, information on the mother's age, anthropometry, marital status, education level, and health were collected and recorded in the Case Report Form (CRF). Breastfeeding was assessed using the recommended WHO breastfeeding questionnaire, breastfeeding observation aid (WHO, 2010b), and a 14‐day recall. Additional data on the infant's home, socio‐economic, and social support environment and breastfeeding history were also collected during hospitalization and recorded in the CRF. Participants phone numbers and village descriptions were collected to facilitate tracing infants for follow‐up.

### 2.6 Hospitalization

Each morning during admission, infants were seen by the paediatric clinician and by the PN. Inpatient treatment including antibiotics and supplementation with dilute F100 when breast milk was initially insufficient was given as per WHO recommendations. Information specific to the breastfeeding support process including breastfeeding challenges, breast conditions, feeds volume and times, and weight were collected daily by the BFPS and recorded in a specifically designed lactation management form. Weight velocity was calculated every morning by the PN and recorded in the lactation management form developed by the study team.

### 2.7 Discharge

Infants were ready for discharge once the clinical condition had been resolved and were observed to have a weight velocity of >5 g/kg/day on breastmilk only for three consecutive days. Clinicians were requested to consult with the PN before discharging a participant and on discharge participants were provided with a return‐date, 2‐week postdischarge, for their first follow‐up visit at KCH.

### 2.8 Follow‐up

Infants discharged alive were followed up at the second, fourth, and sixth weeks after discharge. Mothers were asked to return to the hospital for follow‐up. Mothers who failed to turn up were traced by phone and asked if they would like to have their visit rescheduled. Mothers who could not be traced by phone were followed up at home using the village description collected at discharge. At each postdischarge visit, breastfeeding status, infant growth, and any history of hospital readmission, morbidity, or mortality were ascertained.

### 2.9 Statistical analysis

Data recorded in the CRF and the lactation management forms were entered into a REDCap database. Analysis was undertaken using STATA 15 software (Stata corp. Tx, USA). Descriptive analysis includes proportions and where applicable their respective 95% confidence intervals and median with interquartile ranges (IQRs).

### 2.10 Ethical considerations

Scientific and ethical approval for implementation of this study was granted by the Kenyan national scientific and ethical review unit (SERU) under study number KEMRI/SERU/CGMR/050/3285. Informed written consent was obtained from each participating mother before recruitment into the study. The study was rigorously monitored by trained internal clinical trial monitors at initiation, midterm, and at close‐out.

## 3 RESULTS

### 3.1 Participants

**Figure 1:**
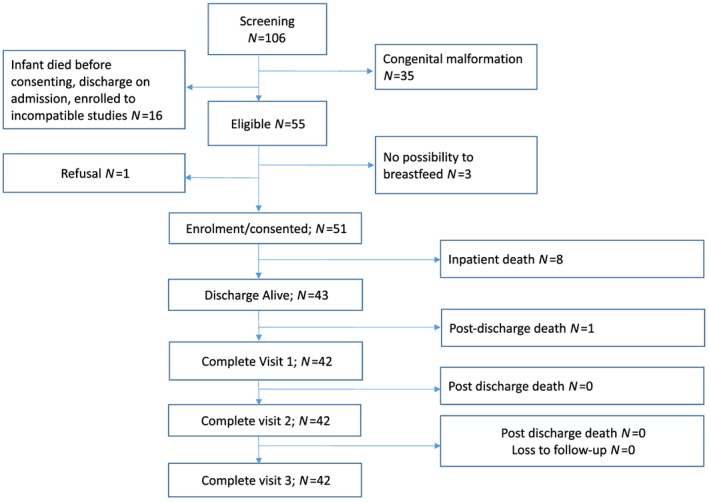
Flow chart of study participants

One hundred and six infants fitted the initial anthropometric criteria. Thirty‐five (33%) were excluded due to congenital malformation. Of the remainder, 3 (6%) had no possibility to breastfeed and another 16 (15%) were either discharged or died on admission. One participant declined to participate **(**Figure 1 **)**. Of the 51 infants included, median age was 2 months (IQR 1 to 3 months), 22 (43%) were reported to have been born with low birth weight (birth weight < 2.5 kg), and 11 (%) had unknown birth weight (Table 1). Thirty‐eight (75%) infants were reported to have been born in hospital. At admission, six (9%) infants with a body length of below 45 cm had incalculable WLZ scores**.** Pneumonia was the commonest illness at admission (76%) followed by diarrhoea (29%). HIV exposure was 10%.

**Table 1:** Descriptive characteristics of study participants

Maternal demographics	Median (IQR)	
Age (years)	28 (23 to 33)	
Parity (children)	3 (IQR 1 to 5)	
BMI	20.9 (IQR 18.7 to 23.2)	
MUAC (cm)	24.3 (IQR 23.1 to 26.4)	
Maternal household characteristics	Number (%); (*N* = 51)	
Employment status (labourer):	18 (35%)	
Education level (none):	16 (31%)	
Marital status (married)	47 (92%)	
Living in extended family	29 (57%)	
Family head (spouse)	21 (41%)	
Financial support (biological father)	43 (84%)	
Occupation of financial support (Labourer)	27 (53%)	
Maternal mental health status (PHQ 9)	Discharge; (*N* = 42)	Day 28 visit; (*N* = 41)
Mild, moderate, severe depression	5 (12%)	3 (7%)
Unknown/incomplete	13 (31%)	2 (5%)
Maternal main support network	Hospitalization; (*N* = 42)	Day 28 visit; (*N* = 41)
Caregiver's mother (maternal grandmother)	20 (48%)	26 (63%)
Spouse	15 (36%)	7 (17%)
Caregiver's mother in law (paternal grandmother)	4 (10%)	7 (17%)
Type of support offered to mother	At hospital	At home
Moral support (words of encouragement)	28 (67%)	36 (88%)
Visitation (face to face)	4(10%)	
Financial (money)		4 (10%)
Infant demographics	Median (IQR)	
Age (days)	52 (36 to 68)	
Length of stay (days)	7 (5 to 11)	
Admission weight (kg)	3.01 (2.25 to 3.7)	
Admission length (cm)	50 (46.3 to 53.0)	
Admission MUAC (cm)	10 (8.2 to 11.0)	
Infant birth history	Number (%); (*N* = 51)	
Hospital birth:	38 (75%)	
Low birth weight:	22 (43%)	
BCG:	43 (84%)	
Infant clinical condition	Number (%) (*N* = 51)	
Pneumonia	38 (74%)	
Diarrhoea	15 (29%)	
HIV exposure	5 (10%)	

Abbreviations: IQRs: interquartile ranges; MUAC: mid‐upper‐arm circumference.

The median age of the mothers was 28 years (IQR 23 to 33 years); 31% of them had no formal schooling, and 35% worked as casual labourers. Their median body mass index was 20.7 (IQR 18.3 to 23.5), and median MUAC was 24.5 cm (IQR 23.1 to 26.4). Maternal mental health status measured using the PHQ 9 test recorded 12% any depression at discharge. The proportion diminished to 7% by day 28 follow‐up. Majority of mothers reported receiving support during hospitalization and follow‐up mainly from the infant's maternal grandmother. The main type of support being moral support, that is, sharing words of encouragement.

### 3.2 Infant feeding

**Figure 2:**
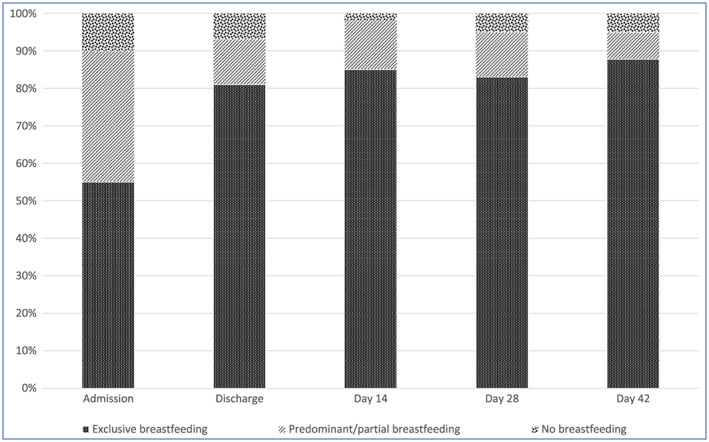
Breastfeeding status from admission to day 42 visit

**Figure 3:**
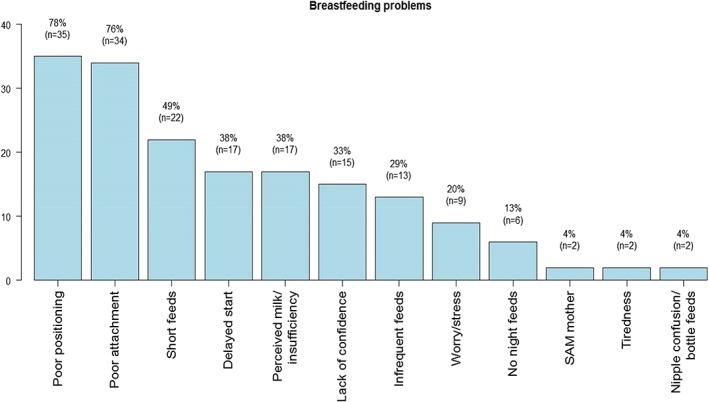
Observed breastfeeding challenges at admission

At admission, 55% of the infants were reported to be exclusively breastfeeding and 33% predominantly breastfeeding **(**Figure 2 **)**. At admission, about 5 of the 51 infants (10%) had complete lactation failure (no breastmilk consumption). Reasons for complete lactation failure included separation at birth, choice to not breastfeed, severely ill, and severely malnourished mothers**.** Consumption of prelacteal feeds was reported in 40% and mixed feeding in 47% of enrolled infants. Common feeds given in both scenarios included plain water, sweetened water, and diluted cow's milk. Mothers were observed to have multiple breastfeeding challenges. Poor positioning and attachment were observed in 78% and 76% of the mothers, respectively, and having had a delayed start to breastfeeding and perceived milk insufficiency was reported in 34% of the mothers. Other challenges such as infrequent feeding and infants not feeding at night were reported among few mothers (Figure 3).

### 3.3 Infant morbidity and mortality

Eight of the 51 infants (16%) died while in hospital. Infants who died had a median birth weight of 2.7 kg (IQR 2.5, 3.0) and a hospital stay of 6 days (IQR 4, 14). Of the 43 infants discharged alive, one (2.3%) died in the first 14 days of follow‐up. There was no loss‐to follow‐up. During follow‐up, 9/42 (21.4%) infants were reported to have been unwell in the first 28 days after discharge. Common ailments included fever, cough, and diarrhoea. Of the nine, five (12%) were reported to have been re‐admitted to hospital.

### 3.4 Meeting the WHO nutritional rehabilitation discharge criteria

**Figure 4:**
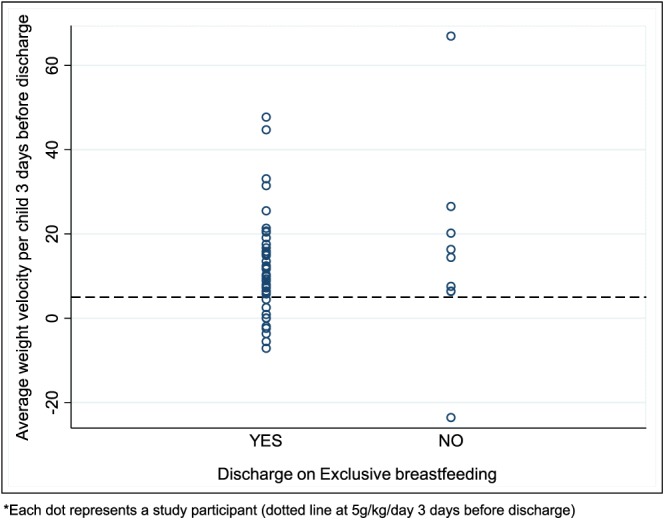
Average weight velocity of participants 3 days before discharge

Of the 43 infants discharged alive, 35 (81.4%) were exclusively breastfeeding by the time of discharge. The remaining 8/43 (18.6%) infants were discharged without the reestablishment of EBF. The median discharge weight velocity for all infants in the last 3 days before discharge was 14.5 g/kg/day (IQR 3.9 to 25.3). However, of all infants discharged alive, only 29/43 (67.4%) were discharged having attained a weight velocity of >5 g/kg/day on breastmilk alone for three consecutive days as recommended by WHO guidelines **(**Figure 4 **)**.

### 3.5 Infant growth

**Figure 5:**
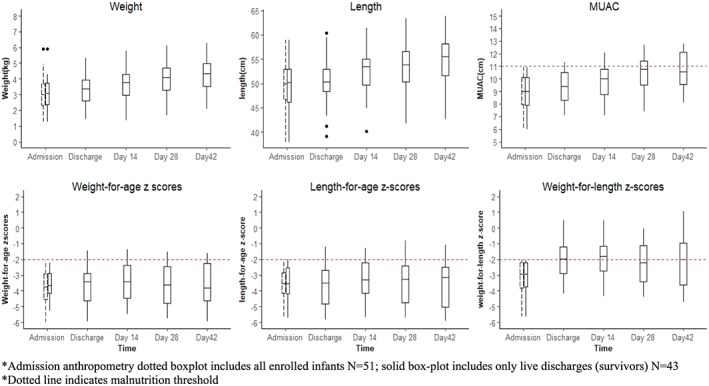
Summary of anthropometric measures in all visits

The average admission, discharge, and postdischarge anthropometric summaries are provided in Figure 5. The median weight, length, and MUAC of the infants gradually increased from 3.01 kg, 50.0 cm, and 9.0 cm, respectively, at admission to 4.32 kg, 55.5 cm, and 10.6 cm, respectively, at the day 42 post discharge visit. The WLZ and WAZ scores also gradually increased between admission and day 14 visit: from −3.67 to −1.69 and −4.12 to −3.83 *Z* scores, respectively. Thereafter, WLZ declined to −2.18 and WAZ stagnated at −3.82 by day 42 visit. Length for age *Z* scores did not improve during the study period.

## 4 DISCUSSION

We present the first published research evidence on the implementation of the 2013 WHO guidelines for the management of ill malnourished infants under 6 months in a public hospital in a resource poor setting. We found that BFPS are able to operate in an inpatient setting to facilitate the effective implementation of the 2013 WHO nutritional rehabilitation guidelines. Using the BFPS strategy, the proportion of infants exclusively breastfeeding increased from 55% at admission to 81% at discharge and 67% of all infants discharged alive met the recommended discharge criteria. However, during follow‐up, gains in WLZ and WAZ were generally not sustained beyond 2 weeks after discharge.

In this pilot study, infants without a possibility to breastfeed made up only a small proportion (6%) of infants u6m admitted with an infection and acute malnutrition. This finding suggests that focusing on breastfeeding for the management of ill, malnourished infants is likely to be feasible for the majority of the targeted infants u6m. However, we also found that a considerable proportion (35%) of identified infants had some form of congenital malformation that would require specialized clinical and feeding care beyond breastfeeding support currently recommended in the guidelines. Being able to distinguish these infants at admission and accounting for them in burden estimates will give a clearer picture of the proportion of infants u6m who may benefit from the current guidelines and build a case for providing a clearer alternative for those that might not.

Applying the current guidelines our pilot study found that almost half (43%) of the ill, malnourished infants had a history of small size at birth either from being low birth weight, prematurity or small for gestational age. This finding further emphasizes the importance of size at birth as a contributing risk factor for malnutrition in early infancy. Studies from birth cohorts have found that the risk of death associated with low birth weight persists months after birth (Mwangome et al., 2019) suggesting that infants with a history of small size should continue to be targeted with interventions throughout infancy to improve growth and lower their risk of mortality.

In this study, six infants had incalculable WLZ scores because their admission length was <45 cm and the charts do not go below this length. Their identification and inclusion into the study were based on alternative anthropometric measures such as MUAC and WFA. Studies have identified incalculable WLZ as one of the major practical challenges in early identification of malnourished infants (Grijalva‐Eternod et al., 2017). Others have found incalculable WLZ at any age to be associated with mortality (Mwangome et al., 2019). Guidelines need to be reviewed to target highly vulnerable infants using alternative anthropometry. Research has shown that around the age of vaccination, MUAC and WAZ are better associated with mortality than WLZ at the same age (Mwangome et al., 2017). Our results indicate a need to consider MUAC and/or WAZ as alternatives to WFL in identifying growth failure in under 6 months.

In most settings, breastfeeding support offered during hospitalization is unstructured, unsupervised, and undocumented. In this study, the employment of dedicated personnel (the BFPS) as well as the development and application of a SOP and the introduction of a lactation management record form provided structure, accountability, and objectivity to the breastfeeding support strategy. This combination of structure, together with well‐trained and dedicated staff and the application of assessment and evaluation tools, allowed for the effective implementation of the WHO, 2013 guidelines. Evidence suggests that structured breastfeeding support programmes are more successful than unstructured programmes (Beake, Pellowe, Dykes, Schmied, & Bick, 2011) and findings from a review of a similar intervention, the Baby Friendly Hospital Initiative, acknowledge that the availability of well‐trained and dedicated staff is key to the success of breastfeeding interventions offered within a hospital setting (Aryeetey & Dykes, 2018). Our results suggest that a structured BFPS strategy can successfully address the challenges encountered in the implementation of the 2013 WHO guidelines for the rehabilitation of malnourished infants u6m. However, the BFPS in this study were employed under trial conditions and their integration into health system needs to further be explored. The experiences and impact of applying the BFPS strategy on the mothers and other health workers are reported in follow‐up manuscripts.

Of the 81% infants discharged on EBF, only 67% had attained the breastfeeding discharge criteria recommended in the guidelines; growth velocity of >5 g/kg/day on breastmilk alone for three consecutive days. These results indicate that not all infants discharged on EBF would be growing at an acceptable rate on breastmilk only. An important reason for discharge before reaching the growth velocity discharge criteria was concern among clinicians of the risk of cross‐infection associated with a long hospital stay. Reestablishing EBF is a time‐consuming process. In this study the median length of hospital stay was 7 days, which was shorter than what has been reported elsewhere (Kayhan‐Tetik, Baydar‐Artantas, Bozcuk‐Guzeldemirci, Ustu, & Yilmaz, 2013; Wilkinson & Isanaka, 2009). Additional days in hospital may have produced better breastfeeding outcomes; however, these potential benefits will have to be weighed against the potential risk of acquiring hospital antimicrobial‐resistant bacterial infections and the cost of longer hospitalization to the family. Future studies need to explore strategies for sustained structured breastfeeding support outside the hospital environment to maximize on the potential benefit of continued breastfeeding among recovering malnourished infants.

This is the first study to gather follow‐up anthropometric data of infants u6m recovering from malnutrition as previous studies on inpatient treatment of malnutrition among u6m did not follow‐up infants after discharge (Corbett, 2000; Seema Patwari & Satyanarayana, 1997; Singh et al., 2013). During postdischarge follow‐up, infants in our pilot study experienced a gradual increase in weight and MUAC, but by 6 weeks after discharge, this was not enough to meet the criteria for full nutritional recovery as defined by MUAC or WAZ or WLZ scores. For example, anthropometric increase in WLZ did not continue beyond 2 weeks after discharge. The lack of continued increase in anthropometric *Z* scores after discharge suggests sustained nutritional vulnerability among this particularly vulnerable group. Future studies are needed to identify and test sustained nutritional support approaches that can be applied after discharge to sustain growth of infants u6m recovering from malnutrition. Approaches like the community management of at risk mothers and infants (Emergency Nutrition Network, 2018) that provides health workers with tools to assess, identify, and manage at risk mothers and infants u6m in the community should be assessed for their applicability and their integration into the public health system in low and middle income countries.

### 4.1 Study limitations

The study was implemented during a period of health workers strikes in Kenya (approximately 8 months of strike between 2016 and 2017; Irimu et al., 2018). This affected the study recruitment rate and resulted in a small number of participants in the final analysis. Even so, rich quantitative data collected during implementation can be used to inform the direction of research in this area. Secondly, the use of recall methods to evaluate breastfeeding status is associated with recall bias. Future studies should apply more reliable methods such as the use of stable isotope techniques. Lastly, as a pilot, the study implemented a short follow‐up period, hence missing out on data during the feeding transition period when infants turn 6 months old. Future studies should include a much larger cohort of infants to account for outcomes for different subgroups by age, size at birth, and breastfeeding status. Studies should also apply a much longer period of follow‐up at least up to 6 months of age.

## 5 CONCLUSION

Breast feeding peer supporters were able, in an inpatient setting, to apply the 2013 updated WHO guidelines. The use of SOPs ensured standardized application of breastfeeding support tools and procedures. However, lack of continued increase in anthropometric *Z* scores after discharge suggests the need for more sustained approaches.

## CONFLICTS OF INTEREST

The authors declare that they have no conflicts of interest.

## CONTRIBUTIONS

MM, AA, CJ, and JB conceived and designed the study. MM, JK, PG, GM, AT, LK, BS, and NM designed study tools and facilitated training and monitoring of study procedures and data collection. MM, SM, and JB designed database and managed quantitative data analysis and interpretation. MM, JK, and CJ managed qualitative data collection, analysis, and interpretation. MM drafted the initial manuscript. All authors have revised and approved the final manuscript.

## Supporting information


**Table S1**: Introduction to lactation training schedule
**Table S2**: Household characteristics of study participants
**Figure S1**: Summary of steps adopted in the Standard Operation Procedure (SOP)
