# Supplementary material for: Individualized breastfeeding support for acutely ill, malnourished infants under 6 months old
Source: Matern Child Nutr. 2019 Aug 2;16(1):e12868. doi: 10.1111/mcn.12868 (PMC7038891; doi:10.1111/mcn.12868)
Supplement: Supplementary file 1 — Table S1: Introduction to lactation training schedule Table S2: Household characteristics of study participants Figure S1: Summary of steps adopted in the Standard Operation Procedure (SOP) [file MCN-16-e12868-s001.docx]

| **SESSIONS** | **TOPICS** | **REFERENCE MATERIAL** |
| --- | --- | --- |
| Introduction (20min) | Introduction to the IBAMI study | IBAMI protocol |
| Session 1 (45 minutes) | Global strategy on Maternal Infant and Young Children Feeding (MIYCF)  WHO recommendation of management and treatment of SAM in infants under 6 months | Global MIYCF strategy (2007)^[[1]](#footnote-1)^, WHO updated guidelines (2013)^[[2]](#footnote-2)^, National Integrated Management of Acute Malnutrition (IMAM) guidelines (2016)^[[3]](#footnote-3)^ |
| Session 2 (50 min) | National policies on breastfeeding  Breastmilk substitute (BMS) regulation and control act | BMS act^[[4]](#footnote-4)^, MIYCN strategy, MIYCN operational guidelines |
| Session 3 (90min) | Maternal nutrition  Food groups, During pregnancy, During lactation, Family planning | UNICEF counselling cards; Mother child booklet |
| Session 4 (35min) | Importance of breastfeeding/ risk of not breastfeeding | Baby Friendly Community Initiative implementation guideline (BFCI) ^[[5]](#footnote-5)^  Global Health Media Project videos^[[6]](#footnote-6)^ |
| Session 5 (45min) | How breastfeeding works  Anatomy, Reflexes, Oxytocin, Demand feeding | Infant and young child feeding counselling: An integrated course^[[7]](#footnote-7)^  WHO relactation manual^[[8]](#footnote-8)^ |
| Session 6 (120 min) | Counselling skills  Listening and learning’ Building confidence and support | Baby Friendly Community Initiative implementation guideline (BFCI)  WHO relactation manual |
| Session 7 (60 min) | Common breastfeeding difficulties  Crying baby, refusing to breastfeed | Baby Friendly Community Initiative implementation guideline (BFCI) |
| Session 8 (60 min) | Breastfeeding techniques  Attachment, Positioning, Frequency | Global Health Media Project Videos^[[9]](#footnote-9)^  Global issues in local context Videos (Glocal)^[[10]](#footnote-10)^  Infant and young child feeding counseling course^7^ |
| Session 9 (45min) | Expressing breastmilk  Hand washing, Hand expressing, (circumstances, technique, storage, hygiene, warm compress, cup feeding), Breast pump | IMAM  BFCI; Practicum |
| Session 9 (45 min) | Breast conditions that affect breastfeeding and remedies  Inverted, Cracked, engorgement | Use Glocal and Global health breastfeeding videos if available; BFCI |
| Session 10 (120 min) | Infant feeding in special circumstances  -Low birth weight (30min)  - Feeding during illnesses (20min)  (support for lactating mother)  - Infant feeding in the context of HIV/TB (60min)  - Danger signs (convulsion/diarrhoea)  - Infants without possibility to breastfeed | BFCI  IMAM (focus nutritional rehabilitation for under 6 months)  Low birth weight guidelines |
| Session 11 (120 min) | Triaging and critical pathway  Nutritional assessment/diagnosis/z scores  10 steps of management (focus on feeding u6m) | IMAM guidelines  Practicum |
| Session 12 (180 min) | Feeding undernourished child less than 6 months  Supplementary Suckling Technique (SST) when and how?  Tube feeding when and how? | Pediatric protocol  WHO relactation manual |

S Table 1: Introduction to lactation training schedule

| **WASH Variable** | **Proportion** | **ASSET Variable** | **Proportion** |
| --- | --- | --- | --- |
| Has electricity, n (%) | 10(20%) | Home ownership, n (%) |  |
| Sources of drinking water, n (%) |  | Lives in own home | 41(80%) |
| Pipe to dwelling | 4(8%) | Paying rent | 9(18%) |
| Piped water to plot | 2(4%) | Not paying rent | 1(2%) |
| Piped water to neighbor | 17(33%) | Floor material, n (%) |  |
| Stand pipe | 20(39%) | Earth/mud/sand | 35(69%) |
| Protected well | 2(4%) | Cemented/ ceramic tiles | 16(31%) |
| Stream/river/lake/pond/dam | 6(12%) | Main wall of the house, n (%) |  |
| Sources of water for other purposes, n (%) |  | Mud | 33(65%) |
| Pipe to dwelling | 4(8%) | Grass/Makuti | 2(4%) |
| Piped water to plot | 2(4%) | Stone/bricks | 16(31%) |
| Piped water to neighbor | 17(33%) | Sources of Fuel, n (%) |  |
| Stand pipe | 19(37%) | Charcoal | 11(22%) |
| Protected well | 3(6%) | Firewood | 38(75%) |
| Stream/river/lake/pond/dam | 6(12%) | Electricity/gas | 1(2%) |
| Distance to get drinking water, n (%) |  | Separate kitchen room, n (%) | 29(57%) |
| Less than 500m (15 minutes) | 35(69%) | Ownership of electronics, n (%) |  |
| 500m to 2 km (15 -1hr) | 13(25%) | Television/radio | 5(10%) |
| >2km (1-2 hours) | 3(6%) | Mobile phone | 44(86%) |
| Storage of drinking water, n (%) |  | Refrigerator | 2(4%) |
| Closed container | 3(6%) | Transportation, n (%) |  |
| Open container | 48(94%) | Bicycle | 20(39%) |
| Purifies drinking water, n (%) | 4(8%) | Motorcycle | 16(31%) |
| Sharing toilet facility, n (%) | 27(53%) |  |  |
| Toilet facility for house members, n (%) |  |  |  |
| Own pit latrine | 31(61%) |  |  |
| Flush toilet | 7(14%) |  |  |
| Open defecation(bush) | 13(25%) |  |  |
| Practice hand washing, n (%) |  |  |  |
| Before breastfeeding | 17(33%) |  |  |
| After handling child toilet | 29(57%) |  |  |
| Before eating | 50(98%) |  |  |
| Before cooking | 23(45%) |  |  |
| After visiting the toilet | 38(75%) |  |  |

S Table 2: Household characteristics of study participants

^[[11]](#footnote-11)^

Step 1: Evaluation and assessment

Breastfeeding history

- Place of birth, pre-lacteal, mixed feeding, frequency of breastfeeding, breastfeeding on both breasts**^11,^**

Breast assessment

- Breast hygiene, appearance, nipple abnormalities, oxytocin reflex, inflammation, infection, pain**^12^**

Breastfeeding techniques

- Mother healthy, relaxed bonding; infant relaxed, calm, infant roots for breast, positioning, attachment and suckling**^12^**

Diagnose main breastfeeding challenges

- Breastfeeding technique, mother’s factors i.e. perceived milk insufficiency, baby’s condition i.e. abnormality or mother’s physical health i.e. pregnant **^12^**

Recommend Lactation plan

- Prioritize package of care i.e. how breastfeeding works, technique, hygiene, hand expression etc

- Supplement with DF100**^13^**

Step 2: Review to exclusive breastfeeding

Full application of the lactation plan

- Briefing from BFPS and records from BFPS daily log of activities

Increased milk output

- Briefing from BFPS, records from daily feeding chart, mother’s testimony, infant off DL 100

Consistent weight gain

- Briefing from BFPS and records from daily feeding and weighing charts, mother’s testimony

Recommendation

- Revised lactation plan to be reviewed in 3 days

-Observe growth on breastmilk only for 3 days

Step 3: Review for discharge

Sufficient weight gain (>5g/kg/day) on breastmilk alone for 3 consecutive days

- Report from BFPS, records from daily feeding chart, infant good appetite

Caregiver confident with breastmilk output

- Report from BFPS, mother’s testimony and reports breastmilk to be sufficient for infant

Lactation failure: Intensive support for 14 days

- Failure to graduate to step 3 in 14 days

S Figure 1: Summary of steps adopted in the Standard Operation Procedure (SOP)^[[12]](#footnote-12)^^[[13]](#footnote-13)^

1. Ministry of Health, K. (2007). "National Strategy on Infant and Young Child Feeding 2007 to 2010." from https://extranet.who.int/nutrition/gina/sites/default/files/KEN%202007%20National%20Strategy%20on%20Infant%20and%20Young%20Child%20Feeding.pdf. [↑](#footnote-ref-1)
2. WHO. (2013). "Guideline: updates on the management of severe acute malnutrition in infants and children." from http://apps.who.int/iris/bitstream/10665/95584/1/9789241506328_eng.pdf. [↑](#footnote-ref-2)
3. Ministry of Health (2009). National Guidelines for Intergrated Management of Acute Malnutrition, Ministry of Health, Kenya. [↑](#footnote-ref-3)
4. (2012). "THE BREAST MILK SUBSTITUTES (REGULATION AND CONTROL) BILL, 2012." [↑](#footnote-ref-4)
5. Ministry of Health, K. (2016). "Baby Friendly Community Initiative Implementation Guidelines

   ", from https://www.mcsprogram.org/wp-content/uploads/2018/04/BFCI-Implementation-Guidelines.pdf. [↑](#footnote-ref-5)
6. https://globalhealthmedia.org/videos/breastfeeding/ [↑](#footnote-ref-6)
7. WHO and UNICEF (2006). Infant and Young Child Feeding Counselling: An intergrated Course. Geneva, Worls Health Organization. [↑](#footnote-ref-7)
8. Organisation, W. H. (1998). "Relactation: A review of experience and recommendations for practice." from http://www.who.int/maternal_child_adolescent/documents/who_chs_cah_98_14/en/. [↑](#footnote-ref-8)
9. https://globalhealthmedia.org/videos/breastfeeding/ [↑](#footnote-ref-9)
10. http://www.glocalnutrition.com/About [↑](#footnote-ref-10)
11. <http://whqlibdoc.who.int/publications/2010/9789241599290_eng.pdf> [↑](#footnote-ref-11)
12. <http://www.who.int/nutrition/publications/infantfeeding/9789241594745/en/> [↑](#footnote-ref-12)
13. <http://apps.who.int/iris/bitstream/10665/95584/1/9789241506328_eng.pdf> [↑](#footnote-ref-13)
